# Long-Term Followup of Laser *In Situ* Keratomileusis for Hyperopia Using a 213 nm Wavelength Solid-State Laser

**DOI:** 10.1155/2013/276984

**Published:** 2013-03-03

**Authors:** Carmina Franz G. Quito, Archimedes Lee D. Agahan, Raymond P. Evangelista

**Affiliations:** ^1^Department of Ophthalmology and Visual Sciences, University of the Philippines, Philippine General Hospital, Taft Avenue, Manila 1000, Philippines; ^2^Refractive Surgery Service, Manila Vision Correction Center, Ermita, Manila, Philippines

## Abstract

*Purpose*. To evaluate the long-term efficacy, accuracy, stability, and safety of hyperopic laser *in situ* keratomileusis (LASIK) using a 213 nm wavelength solid-state laser. *Methods*. This prospective noncomparative case series consisted of 34 eyes of 17 patients which underwent hyperopic LASIK using a 213 nm solid-state laser (Pulzar Z1, CustomVis) at an outpatient refractive surgery center in Manila, Philippines. The preoperative and postoperative examinations included uncorrected distance visual acuity (UDVA), subjective manifest refraction, corrected distance visual acuity (CDVA), cycloplegic refraction, slitlamp biomicroscopy, and keratometry (*K*). *Main Outcome Measures*. Accuracy, efficacy, stability, and safety of the refractive procedure. *Results*. Mean follow-up was 25.18 ± 13.79 months. At the end of follow-up, 26.47% had a UDVA of 20/20 and 94.12% had a UDVA of ≥20/40. Manifest refractive spherical equivalent (MRSE) was within ±0.50 D of the target refraction in 55.88% and within ±1.0 D in 85.30% of the study eyes. Refractive stability was noted in the 1st postoperative month while hyperopic regression was noted after the 3rd postoperative year. No eye lost more than 2 lines of CDVA. *Conclusion*. Our results show that the 213 nm solid state laser system is safe, effective, accurate, and predictable for the treatment of hyperopia.

## 1. Introduction

Laser *in situ* keratomileusis is approved by the US Food and Drug Administration (FDA) for the treatment of myopia, hyperopia, and astigmatism and has been a popular choice among refractive surgeons for almost 2 decades now [1, 2]. Hyperopic LASIK consists of an annular zone of ablation to cause a relative flattening of the corneal periphery and a concomitant relative steepening of the center (optical zone) to achieve the desired refractive effect.

Early attempts to correct hyperopia included techniques such as hexagonal keratotomy, thermokeratoplasty, keratophakia, and keratomileusis but have met with only limited success. With the advent of the excimer laser technology, reshaping of the corneal surface to a desired contour with submicron precision and reproducibility became a reality [3]. With the prototype ophthalmic excimer lasers, the visual and refractive outcomes of LASIK for hyperopia were less predictable due to challenges arising from the need to use larger ablation diameters and the relative difficulty to deliver uniformly distributed laser energy in ablation zones larger than 6 mm [1]. Newer models have permitted larger ablation diameters and the development of various microkeratomes facilitated the construction of larger corneal flaps [1, 3]. Despite this technology, predictability and stability are markedly reduced with treatment, especially of high levels of hyperopia [4–7].

With the success of excimer lasers, the desire to develop a solid-state laser with an output wavelength of 213 nm was born. This resulted from the problems associated with gas lasers, including the use of the toxic gas fluorine, which is a safety issue in the clinical environment. In recent years, this Nd:YAG laser has gained recognition as a possible alternative to the long-established 193 nm excimer laser system.

In addition to the absence of toxic gas, recent evidences have been discussed and demonstrated the advantages of the solid-state laser over the traditional excimer laser. It has been shown to create a smoother ablation surface by the accurate transfer of energy onto the corneal stroma [8–11]. A smaller spot size of 0.6 mm, which is 2.5 times smaller than a typical excimer laser spot size, contributes less mechanical stress on the cornea causing less damage to the corneal structure [8, 12]. Histopathologic evidence has demonstrated a more favorable response after irradiation with 213 nm wavelength which may provide a more predictable wound healing and refractive response [9]. Furthermore, the 213 nm wavelength solid-state laser has been proven to be less sensitive to corneal hydration, limiting its effect on the final refractive outcome [13].

The purpose of this study was to evaluate the long-term efficacy, accuracy, stability, and safety of laser *in situ* keratomileusis using a 213 nm wavelength solid-state laser for the treatment of hyperopia.

## 2. Materials and Methods

This prospective consecutive case series was done at an outpatient refractive surgery center in Manila, Philippines. This study was evaluated and approved by the hospital ethics review board. All patients with hyperopia who consulted at the center without any evidence of other ocular pathology that might affect the final visual and refractive outcomes were included. 

Informed consent was signed by each patient prior to the procedure. The preoperative and postoperative examinations included uncorrected distance visual acuity (UDVA), subjective manifest refraction, corrected distance visual acuity (CDVA), cycloplegic refraction, slit lamp biomicroscopy, and keratometry (*K*). Snellen chart visual acuities were converted to logMAR notation. Manifest refractions (MR) were converted to manifest refractive spherical equivalent (MRSE). Patients with any of the following conditions were not considered candidates for surgery: suspected ectasia or keratoconus, active ocular disease such as glaucoma and uveitis, significant cataract or retinal pathology, thin cornea (<500 *μ*m) autoimmune disease, diabetes, or any other serious medical conditions. Patients who are engaged in sports and military activities, breastfeeding and pregnant women, and those who did not consent for the procedure were also excluded from the study. Other exclusion criteria are previous refractive and intraocular surgery and unrealistic expectations.

Two surgeons (A. L. D. Agahan, R. P. Evangelista) performed the laser procedures from September 2006 to September 2010. The target for 32 eyes was emmetropia while 2 eyes were overcorrected to −1.50 D beyond the target correction. Preoperatively, all patients received 2-3 drops of topical Proparacaine and 1 drop of povidone-iodine 5% solution. A lid speculum was inserted to provide adequate exposure. The suction ring was applied to the limbus and a 9.5 mm corneal flap was created using the Hansatome microkeratome (Bausch and Lomb Surgical). The flap was reflected and dried with Weck-Cel sponge. The ablation was then carried out using the Pulzar Z1 213 nm wavelength solid-state laser (CustomVis) with centration based on the line of sight (line joining the point of fixation to the center of the pupil). The ablation profile had an annular shape with 5.5 mm optical zone and 8.0 mm treatment zone. 

After the ablation was completed, the corneal flap was repositioned and the edges were dried with Weck-Cel sponges. The retractor was removed and one drop of Prednisolone Acetate 1% was given. The patients were instructed to instill Moxifloxacin 0.3% 1 drop 4x/day, Prednisolone Acetate 1% 1 drop 4x/day, and Carboxymethylcellulose 1 drop 4x/day and were discontinued after 2 weeks. Follow-up visits were at 1 day, 1 week, 1, 3, and 6 months, and 1, 2, 3, and 4 years. Examination included UCVA, CDVA, manifest and cycloplegic refraction, slit lamp examination, applanation tonometry, and keratometry. 

Outcome measures were accuracy (postoperative MRSE, attempted versus achieved correction), efficacy (postoperative UDVA and CDVA compared with preoperative CDVA), stability (average preoperative and postoperative MRSE at each follow-up visit), and safety (change in Snellen lines of CDVA). Other outcome measures were flap- and ablation-related complications and pre- and postoperative keratometry readings. 

## 3. Results

### 3.1. Demographics

Thirty four eyes of 17 patients underwent LASIK for hyperopia.  Table 1 shows the baseline characteristics and demographics of all patients included in the study. All patients had bilateral laser treatment. All eyes were seen at least 6 months postoperatively. Twenty-eight eyes were followed up for at least 1 year, 20 eyes for at least 2 years, 14 eyes for at least 3 years, and 6 eyes for 4 years. Mean followup was 25.18 ± 13.79 months.

### 3.2. Visual Acuity

**Figure 1:**
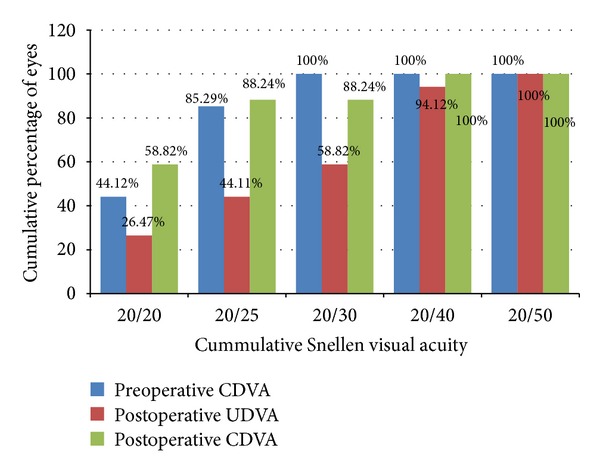
Efficacy of hyperopic LASIK at the last follow-up visit. Cummulative percentage of eyes with UDVA and CDVA at each Snellen line of vision.

The mean UDVA improved from logMAR 0.6 ± 0.25 (~20/80) preoperatively to logMAR 0.17 ± 0.13 (~20/30) at the last followup examination.  Figure 1 shows the cumulative UDVA and CDVA after LASIK compared with the preoperative CDVA. At the last follow-up visit (mean follow-up: 25.18 ± 13.79 months), 9 eyes (26.47%) can see 20/20 without correction and 20 (58.82%) with correction. Likewise, 32 eyes (94.12%) can see 20/40 or better without correction and all eyes can see 20/40 or better with correction. The postoperative UDVA of 17 eyes (50%), 25 eyes (73.53%), and 34 eyes (100%) were within 1, 2, and 3 lines of the preoperative CDVA, respectively.

### 3.3. Accuracy

**Figure 2:**
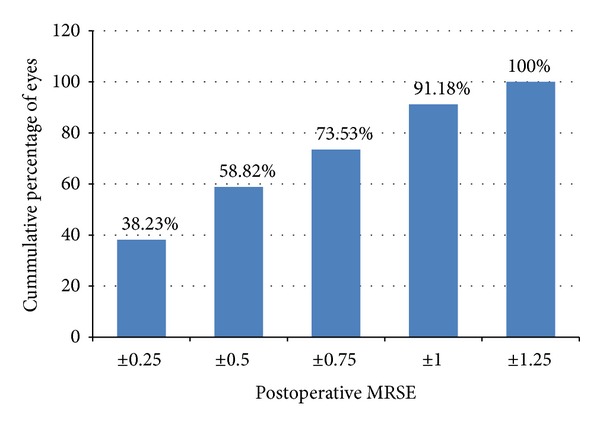
Manifest refractive spherical equivalent (MRSE) at the last follow-up visit (cumulative percentage).

**Figure 3:**
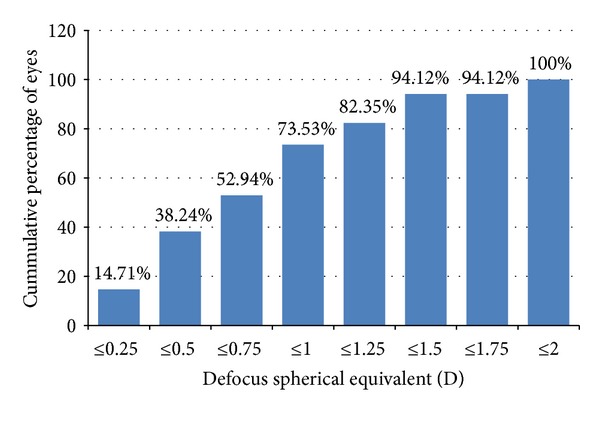
Postoperative defocus spherical equivalent (cumulative percentage).

At the end of the followup, the postoperative MRSE was within ±0.50 D in 20 eyes (58.82%) and within ±1.00 D in 31 eyes (91.18%). None of the eyes had an MRSE beyond 1.25 D of emmetropia (Figure 2). The postoperative defocus spherical equivalent was within 0.50 D in 13 eyes (38.24%), within 1.0 D in 25 eyes (73.53%), and within 1.5 D in 32 eyes (94.12%). No eye had a defocus spherical equivalent of greater than 2 D at the end of followup (Figure 3).

**Figure 4:**
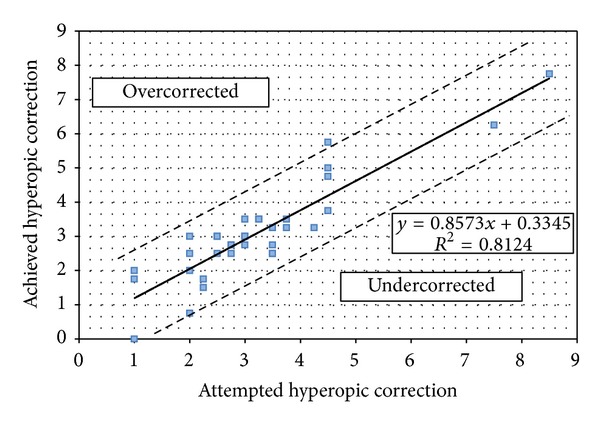
Accuracy of hyperopic LASIK at the last follow-up visit.

**Figure 5:**
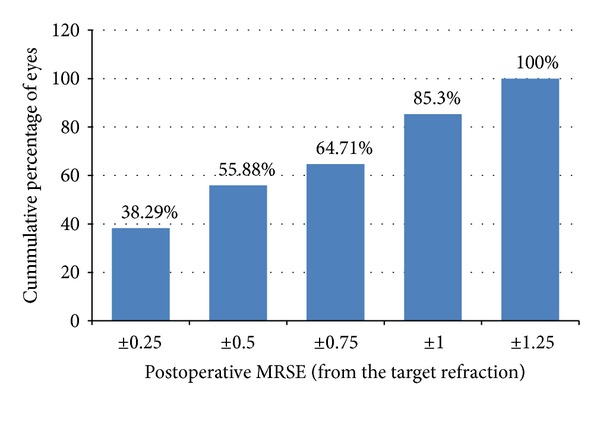
Manifest refractive spherical equivalent (MRSE) at the last follow-up visit (cumulative percentage, with respect to the target refraction).

The mean attempted hyperopic correction was 3.22 ± 1.55 D and the mean achieved hyperopic correction was 3.10 ± 1.48 D. These were shown to be correlated (*R* ^2^ = 0.812) with 19 eyes (55.88%) within 0.50 D of the target refraction and 29 eyes (85.30%) within 1.00 D. No eye had a final MRSE >1.25 D from the target refraction (Figure 5). The linear regression equation in Figure 4 also shows a general tendency towards undercorrection (slope = 0.857, intercept = 0.334).

### 3.4. Stability

**Figure 6:**
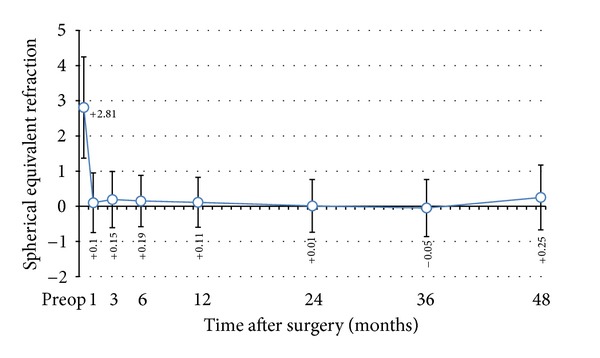
Stability of hyperopic LASIK at 4 years.

The mean MRSE was relatively stable throughout followup (Figure 6). Refractive stability was attained at the first postoperative month and the mean residual refractive error remained relatively stable at each follow-up visit. There was regression of 0.3 D after the third postoperative year with an average of 0.025 D per month.

### 3.5. Safety

**Figure 7:**
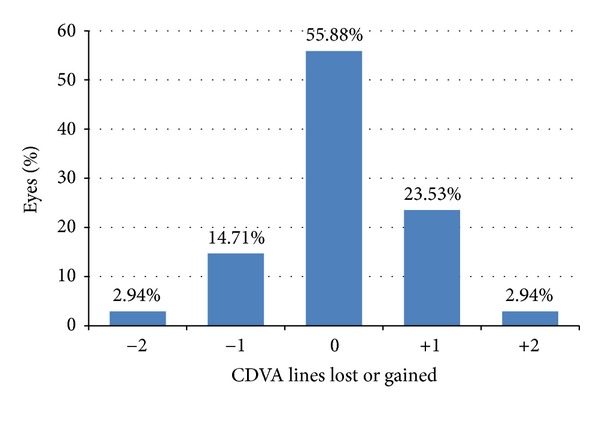
Percentage of eyes that lost or gained lines of CDVA at the last follow-up visit.

**Figure 8:**
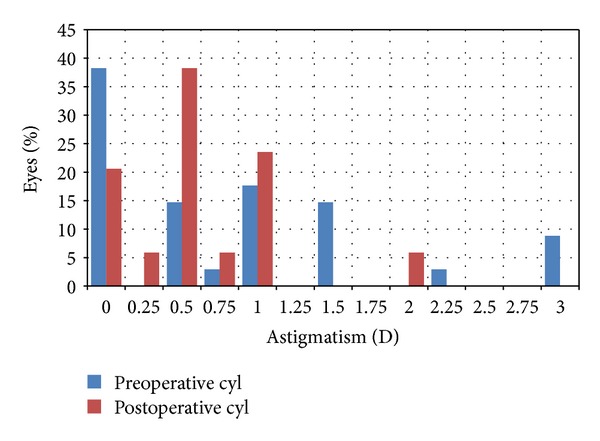
Preoperative and postoperative astigmatism.

Figure 7 shows the number of CDVA lines lost or gained. More than half of the eyes (19 eyes, 55.88%) achieved a postoperative CDVA equal to the preoperative CDVA. No eye lost more than 2 lines of CDVA.  Figure 8 shows the percentage of eyes with various amounts of astigmatism in the preoperative and postoperative period. The mean preoperative cylinder was −0.82 ± 0.92 D and the mean postoperative cylinder was −0.60 ± 0.50 D. The mean change in cylindrical power was −0.22 ± 0.9 D (range: −3 D to +1 D) at the end of the followup. The mean induced astigmatism was 0.66 ± 0.23 D greater than the baseline value in 11 eyes. No eye developed astigmatism greater than 1.0 D. 

The mean keratometry (*K*) value was 42.81 ± 1.81 D preoperatively and 45.53 ± 2.01 D postoperatively. The mean difference between the preoperative and postoperative *K* value was 2.71 ± 1.79 D. The mean difference between *K*1 and *K*2 was 1.23 ± 0.66 D preoperatively and 1.04 ± 0.47 D postoperatively. Based on the *K* values, the mean change in cylindrical power was −0.19 ± 0.83 D (range: −2.25 to +1.25 D) and the mean induced astigmatism was 0.6 ± 0.32 D at the end of the followup. 

No eye had any intraoperative, early, or late postoperative complications. 

## 4. Discussion

The concept of lamellar corneal surgery to alter the refractive state of the eye was first reported by Barraquer in 1949 [3]. He suggested the possibility to manipulate the curvature of the air/tear film interface by adding or removing corneal tissue. It was developed to correct high myopic and hyperopic refractive errors. This concept has undergone a long history of evolution, the most recent of which is the laser *in situ* keratomileusis. This technique combines the wound healing advantages of lamellar procedures with the precision of excimer laser. Several reports have been published regarding its predictability and stability especially for low-to-moderate hyperopic refractive errors [2, 5, 7, 14–19].

The success of the excimer laser technology has led to the emergence of another laser technology, an Nd:YAG laser with an output wavelength of 213 nm. It has been acknowledged as a substitute for the excimer laser because it eliminates the use of the toxic gas fluorine, thus increasing its safety profile.

Our study evaluated the long-term efficacy, accuracy, safety, and stability of laser *in situ* keratomileusis using a 213 nm solid-state laser for the treatment of hyperopia. Thirty-four eyes of 17 patients with preoperative MRSE ranging from +0.75 D to +7.00 D (+2.81 ± 1.44 D) were included. All eyes underwent LASIK using a 213 nm solid-state laser with target refraction ranging from emmetropia up to 1.50 D of overcorrection and were followed up to 4 years postoperatively (mean followup: 25.18 ± 13.79 months).

At the last visit, 26.47% of eyes attained a UDVA of 20/20 and 94.12% had a UDVA of 20/40 or better. These levels coincide with reports by Zadok et al. [2] (≥20/20, 29.3%; ≥20/40, 85.7%), Desai et al. [5] (≥20/20, 34.1%; ≥20/40, 87.8%), and Göker et al. [19] (≥20/20, 14.81%; ≥20/40, 66.66%). However, levels reported by Cobo-Soriano et al. [7] (≥20/20, 46.8%; ≥20/40, 93%), Waring et al. [15] (≥20/20, 70.1%; ≥20/40, 99.3%), and Nepomuceno et al. [20] (≥20/20, 44.4%; ≥20/40, 88.9%) were better than those achieved in our study. The low proportion of eyes having a UCVA of 20/20 or better at the end of followup was attributed to the relatively significant number of eyes having preoperative astigmatism of ≥0.50 D (0.5 D: 14.71%, 0.75 D: 2.94%, 1.0 D: 17.65%, 1.5 D: 14.71%, 2.25 D 2.94%, 3.0 D 8.82%). Two of the study eyes had a preoperative MRSE of +7.0 D and at this higher degree of hyperopia, the result of LASIK becomes less predictable [2, 5, 7, 16, 17]. This could also be contributory to the lower percentage of eyes having a final UDVA of 20/20.

In our study, the mean attempted hyperopic correction was 3.22 ± 1.55 D and the mean achieved hyperopic correction was 3.10 ± 1.48 D. Linear regression analysis showed a good correlation between the attempted and achieved amount of hyperopic correction with *R* ^2^ value of 0.812. MRSE at the last follow-up visit of 55.88% and 85.30% of the study eyes was within ±0.50 D and ±1.0 D of the intended MRSE, respectively, which reflects the refractive accuracy of hyperopic LASIK using the 213 nm laser. This is in accordance with other studies using the conventional excimer laser [2, 4, 5, 7, 14, 15, 18–21]. The mean difference between the preoperative and postoperative *K* values of 2.71 D, which is close to the attempted hyperopic correction of 3.22 D, further mirrors its accuracy in terms of ablation profile. In addition, none of the study eyes required retreatment, which ascertains the accuracy even more.

Refractive stability was noted at the first postoperative month. Hyperopic regression of 0.3 D was observed between the third and fourth postoperative year with an average of 0.025 D per month. Other published works reported refractive stability after the first month [14], at 3 months [17], and at 6 months [15, 22]. Regression was also noted at various postoperative periods, starting at 1 month [14], 3 months [15], and 1 year [5, 16]. In the Beaver Dam Eye Study [23], the mean hyperopic shift over a 5-year period was +0.12 D in a population consisting of individuals aged 43–84 years. Most of the hyperopic change occurred in those younger than 65 years, with a mean positive shift of 0.22 D. Likewise, in the Blue Mountains Eye Study [24] where participants of 49 years or older were enrolled, the mean hyperopic shift over a 5-year period was +0.19 D. The largest hyperopic change was noted in persons aged 49 to 54 years at baseline, recorded at +0.41 D. In those younger than 65 years, the mean positive change was 0.36 D. Since our study consisted of patients younger than 65 years, the regression that occurred during the 4-year period reflects the normal age-related physiologic change in refraction.

Zadok et al. [2] reported complications such as free cap, irregular cut, and irregular astigmatism with 1 eye losing 3 lines of BCVA and 2 eyes losing 2 lines of BCVA, and all were in the high hyperopia group. In our study, more than half (55.88%) had no change in CDVA throughout followup. No complications were encountered during and after the procedure in all study eyes. One eye with preoperative MRSE of +7.0 D lost 2 lines of CDVA. The loss of CDVA lines observed could be due to the high amount of corneal steepening which induced significant degree of optical aberration leading to degradation of image quality. 

The predictability of high hyperopic refractive errors compared with myopic refractive errors of similar degree is much poorer. It was determined that the use of a larger optical and treatment zone offers good predictability and refractive stability for higher degrees of hyperopia by diminishing undercorrection and regression associated with a smaller effective optical zone [16]. Although the upper limit of hyperopic LASIK is frequently set at +5.0 D [1], we do not have enough data to conclude the outcome of this study for higher degrees of hyperopia. 

The incidence of induced astigmatism is relatively higher than previous studies [16, 18], but this did not affect the postoperative CDVA. Possible causes of induced cylinder in LASIK are alteration of the hinge placement and change in the thickness and shape of the lamellar flap [25]. In our study, no correlation was seen between the hinge (placed superiorly) and the axis of the induced cylinder.

We used an optical zone of 5.5 mm with a treatment zone of 8.0 mm which is slightly smaller than the currently recommended larger optical and blend zones. Argento and Cosentino [22] demonstrated better accuracy and stability with the use of optical zones larger than 5.9 mm evidenced by superior visual acuity and lower residual refractive error compared to smaller optical zones. Davidorf et al. [26] likewise compared the ablation profiles of 5 × 9 mm, 5.5 × 8.5 mm, and 6 × 9 mm. He noted a 2.5% increase in spherical equivalent correction for every 0.5 mm increase in optical zone size resulting in a tendency toward undercorrection and overcorrection using a 5 × 9 mm and a 6 × 9 mm ablation profile, respectively. He favored the use of a larger inner zone which should produce a lager functional optical zone. The percentage of 1-line BSCVA loss was the lowest in the 6 × 9 mm group showing a better safety profile. Lastly, El-Helw and Emarah [27] concluded that increasing the optical zone diameter is more predictable and stable in the correction of hyperopia. In our study, accuracy and safety were similar to previous studies wherein larger optical zones were used. The rate of hyperopic regression was not intensified by the use of a smaller OZ, contrary to the mentioned reports.

The present study used a 213 nm solid-state laser which has gained popularity because of its good safety profile. The fundamental wavelength of this laser is 1064 nm, and 3 nonlinear optical crystals can be used to produce the fifth harmonic wavelength of 213 nm. *In vivo* studies have shown that the 213 nm laser provides advantages in the form of a more controlled ablation site and better stability in cell numbers, implying a more predictable ablation outcome. *In vitro*, higher levels of superoxide dismutase (SOD) in corneal keratocytes irradiated with 213 nm suggest a better endogenous protection against free radicals induced by laser surgery [9]. Enucleated rabbit eyes that underwent myopic PRK using both excimer laser and solid-state laser systems showed a smooth ablation surface with no edema or distortion to adjacent corneal tissue, indicating absence of thermal damage [10].

The combination of an extremely small spot (0.6 mm), a uniform intensity beam distribution, a fast pulse rate, and an ultrafast tracking/scanning system contribute to the accuracy of this laser system [11]. The fact that the 193 nm laser system is more sensitive to corneal hydration and environmental humidity is also well known. Absorption at the 213 nm wavelength is 1 to 4 times less sensitive to corneal hydration, thus the refractive correction becomes less sensitive to changes in corneal hydration [13].

The 213 nm solid-state laser system does not require the use of fluoride gas, making it a safer alternative to the 193 nm excimer laser. The laser system has also been shown to be efficient at producing less heat with a maximum temperature of 38.4° generated compared with that of the excimer laser, which is at 52.9°. It operates on a standard wall current without special wiring requiring a lower maintenance and running cost [28].

The study's small sample size, the wide variability in the amount of hyperopia, and the difference in the period of followup are its major limitations. Additionally, corneal topographic changes, patient satisfaction, and quality of vision are also important parameters to be taken into consideration.

In conclusion, our results show that the 213 nm solid-state laser system is safe, effective, accurate, and predictable for the treatment of hyperopia.

## Figures and Tables

**Table 1:** Demographics and baseline characteristics.

Parameter	Value
Patients/eyes (*n*)	17/34
Male/female (*n*)	7/10
Age (y)	
Mean ± SD	45.35 ± 11.72
Range	22 to 63
Preoperative UDVA (logMAR)	
Mean	+0.6 (20/80)
Range	+0.18 to +1.0 (20/30 to 20/200)
Preoperative CDVA (logMAR)	
Mean	+0.1 (20/25)
Range	0.0 to +0.18 (20/20 to 20/30)
Preoperative MRSE (D)	
Mean ± SD	+2.81 ± 1.44
Range	+0.75 to +7.00
Preoperative astigmatism (D)	
Mean ± SD	−0.82 ± 0.92
Range	0.00 to −3.00
Preoperative cyclorefraction SE (D)	
Mean ± SD	+3.10 ± 1.44
Range	+0.75 to +8.50
Attempted correction (D)	
Mean ± SD	3.22 ± 1.55
Range	1.00 to 8.50
Attempted SE (D)	
Mean ± SD	−0.12 ± 0.37
Range	−1.50 to 0
Followup (months)	
Mean ± SD	25.18 ± 13.79
Range	6 to 46

CDVA: corrected distance visual acuity; MRSE: manifest refractive spherical equivalent; UDVA: uncorrected distance visual acuity.
